# Mechanistic Networks, Cellular Specificity, and Therapeutic Opportunities of Ferroptosis in Ulcerative Colitis

**DOI:** 10.3390/ph19060858

**Published:** 2026-05-29

**Authors:** Jia-Le Yi, Ji-Xiao Zhu, Wei-Feng Huang, Li-Tao Yi

**Affiliations:** 1 Department of Chemical and Pharmaceutical Engineering, College of Chemical Engineering, Huaqiao University, Xiamen 361021, China; m15079499029@163.com; 2 Research Center of Traditional Chinese Medicine Resources and Ethnic Medicine, Jiangxi University of Chinese Medicine, Nanchang 330004, China; zhujx81@sina.com; 3 Department of Gastroenterology and Hepatology, The First Affiliated Hospital of Xiamen University, School of Medicine, Xiamen University, Xiamen 361003, China; hwf0625@xmu.edu.cn

**Keywords:** UC, ferroptosis, intestinal epithelial cells, epitranscriptomics

## Abstract

Ulcerative colitis (UC) is a chronic inflammatory disorder characterized by epithelial barrier disruption, oxidative stress, immune dysregulation, and defective mucosal healing. Recent studies have identified ferroptosis, an iron-dependent form of regulated cell death driven by phospholipid peroxidation, as a key mechanism linking these processes. This review summarizes the current progress in understanding the role of ferroptosis in colitis. Available evidence shows that ferroptosis occurs in both human UC and experimental colitis models, with intestinal epithelial cells representing the best-established target compartment. Recent studies have further expanded this concept to reparative immune cells, particularly type 2 (M2) macrophages, thereby indicating that ferroptosis contributes not only to barrier injury but also to impaired mucosal healing. Mechanistically, colitis-associated ferroptosis is governed by interconnected networks involving solute carrier family 7 member 11 (SLC7A11)/glutathione (GSH)/glutathione peroxidase 4 (GPX4) failure, acyl-CoA synthetase long chain family member 4 (ACSL4)-dependent lipid remodeling, iron overload, mitochondrial reactive oxygen species (ROS) amplification, inflammatory signaling, and N6-methyladenosine (m6A)-mediated post-transcriptional regulation. In parallel, microbiota-derived metabolites and dietary factors can either suppress or exacerbate ferroptotic injury. Therapeutically, ferroptosis-targeted strategies, including iron chelation, nutrient-based interventions, natural products, exosomes, and nanoplatforms show promising preclinical efficacy. Overall, ferroptosis provides a connected framework for understanding colitis pathogenesis and provides new opportunities for biomarker development and mechanism-based therapies.

## 1. Introduction

UC is a chronic, relapsing inflammatory bowel disease (IBD) of the colon, and its clinical importance lies not only in persistent mucosal inflammation but also in defective barrier repair, recurrent relapse, and long-term complications. This disease is now understood as a disorder in which epithelial injury, immune dysregulation, microbial imbalance, and oxidative stress converge in the same tissue space [1]. Barrier failure is central to this process, because excessive intestinal epithelial cell death directly increases permeability and permits continuous exposure of the lamina propria to luminal antigens and inflammatory mediators [2,3]. Ferroptosis is highly relevant to this setting because it is an iron-dependent form of regulated cell death driven by phospholipid peroxidation rather than by the classical apoptotic machinery [4]. Its molecular framework is now well established and centers on iron availability, membrane lipid vulnerability, the collapse of antioxidant defense, and GPX4 insufficiency [5,6,7]. This biology is especially meaningful in colitis because the inflamed mucosa is already enriched in reactive oxygen species, cytokine stress, lipid remodeling, and altered iron handling, all of which lower the threshold for ferroptotic injury [8,9,10]. Ferroptosis therefore offers a mechanistic explanation for how oxidative stress is converted into structural epithelial damage, and demonstrates why redox imbalance in colitis is not merely biochemical background noise but a driver of barrier collapse [11,12].

This concept now rests on direct disease-specific evidence. The foundational study that first demonstrated ferroptosis in intestinal epithelial cell death in UC moved the field from theory to pathology [13]. Functional relevance was then strengthened by a study showing that dextran sulfate sodium (DSS) colitis is ferroptosis-associated and can be alleviated by ferroptosis inhibition [14]. The argument became even stronger when colon epithelial ferroptosis itself was proposed as a therapeutic target in UC [15]. Subsequent studies have shown that ferroptosis in colitis is not a single biochemical endpoint but a broader pathogenic network involving inflammatory signaling, nutrient transport, lipid remodeling, and mitochondrial stress, as illustrated by work on signal transducer and activator of transcription 3 (STAT3), solute carrier family 6 member 14 (SLC6A14)/CCAAT/enhancer-binding protein beta (C/EBPβ)-p21-activated kinase 6 (PAK6), carbonic anhydrase 9 (CA9)/stromal interaction molecule 1 (STIM1), and the vitamin D receptor (VDR)–sirtuin 3 (SIRT3)–superoxide dismutase 2 (SOD2)–mitochondrial reactive oxygen species (mtROS) axis [16,17,18,19]. Ferroptosis also matters because it links epithelial injury to iron metabolism and the intestinal ecosystem: iron overload aggravates colitis by modulating ferroptosis and perturbing the microbiota, whereas iron chelation and butyrate both suppress ferroptotic injury while improving barrier-associated phenotypes [20,21,22]. More recent studies have further shown that ferroptosis influences not only acute epithelial damage but also mucosal repair, demonstrating that ferroptosis of reparative M2 macrophages impedes healing, while GPX4-dependent restriction of ferroptosis in intestinal group 3 innate lymphoid cells (ILC3s) helps control intestinal inflammation [23,24]. Taken together, ferroptosis matters in colitis because it unifies epithelial barrier destruction, inflammatory amplification, iron dysregulation, microbiota-metabolite disturbance, and healing failure within one disease framework, while also providing a tractable therapeutic concept whose promise and limitations are now being actively defined [25,26,27,28].

To systematically navigate this expansive biological landscape, this review aims to provide a comprehensive, multi-layered synthesis of the roles, regulatory mechanisms, and translational potential of ferroptosis in UC. The manuscript is organized into six interconnected sections that transition from baseline validation to cellular topography, molecular networks, epitranscriptomic gating, environmental modulation, and therapeutic implementation. In detail, Section 2 evaluates the convergent clinical, biochemical, and ultrastructural evidence establishing the existence of ferroptosis in colitis tissue. Section 3 maps the cell-type specificity of this process, delineating how ferroptosis drives barrier breakdown in the epithelial compartment while crippling resolution pathways in the lamina propria. Section 4 details the core execution networks alongside their upstream transcriptional and mitochondrial regulators. Moving beyond classic protein expression, Section 5 examines the emerging epitranscriptomic landscape, focusing on how m6A RNA methylation machinery determines the threshold for ferroptotic commitment. Section 6 explores the complex crosstalk between host cells and the luminal environment, detailing how microbial postbiotics and dietary substrates either buffer or accelerate lipid peroxidation. Finally, Section 7 critically reviews current pre-clinical anti-ferroptotic therapeutics while addressing their primary pharmacological and translational limitations. Overall, this structured framework highlights remaining challenges and outlines concrete strategies to guide future biomarker development and mechanism-based patient stratification.

## 2. Core Evidence That Ferroptosis Occurs in Colitis

The evidence that ferroptosis occurs in colitis is now supported by convergent findings from human tissues, animal models, epithelial cell systems, ultrastructural analyses, and rescue experiments. In human disease, the earliest decisive advance was the demonstration that ferroptosis participates directly in intestinal epithelial cell death in UC, which moved the field beyond the idea that ferroptosis was merely a secondary oxidative phenomenon in inflamed tissue [13]. This human-oriented interpretation was strengthened by later work identifying colonic epithelial ferroptosis itself as a therapeutic target, thereby reinforcing the fact that ferroptosis is not only present but also biologically important in the diseased mucosa [15]. Subsequent human validation became broader and more mechanistic, showing that UC tissues exhibit reduced GPX4 and increased ACSL4 together with diminished VDR and SIRT3-mediated protection, thus extending the human evidence from classic antioxidant failure to mitochondrial redox dysregulation [19]. Clinical and translational studies have also suggested that ferroptosis-related molecular signatures are detectable at the systems level in UC and may be useful for diagnosis or disease stratification, as shown by work identifying lipocalin 2 (LCN2), dipeptidyl peptidase 4 (DPP4), acyl-CoA synthetase family member 2 (ACSF2), mitofusin 2 (MFN2), and cystathionine beta-synthase (CBS) as ferroptosis-linked candidates in UC [29,30,31,32]. Additional support for a ferroptotic state in human colitis comes from metabolic studies showing that active UC is associated with increased hypoxia-inducible factor-2 alpha (HIF-2α) and divalent metal transporter 1 (DMT1), reduced GPX4, elevated ACSL4, and a more iron-loaded epithelial phenotype, especially in settings linked to bile acid or diet-associated stress [33]. Together, these studies indicate that the human evidence for ferroptosis in colitis is no longer limited to one or two markers, but now includes tissue protein changes, transcriptomic signatures, pathway-level alterations, and clinically relevant molecular stratification.

Animal and cell-based studies provide the experimental depth needed to move from association to causality. A seminal rescue study showed that DSS colitis is accompanied by ACSL4 upregulation, GPX4 and ferritin heavy chain 1 (FTH1) reduction, iron loading, and malondialdehyde (MDA) elevation, and that these abnormalities are attenuated by classical ferroptosis inhibitors and iron chelation, thereby establishing one of the earliest functional links between ferroptosis and experimental colitis severity [14]. This rescue logic has since been reproduced across independent mechanistic systems. GPX4-centered protection was demonstrated in early intervention work showing that ferroptosis suppression ameliorates colitis when GPX4 is restored or when the nuclear factor erythroid 2-related factor 2 (Nrf2)-related antioxidant program is reinforced [34,35,36]. Iron-dependent evidence was further expanded by studies showing that iron overload aggravates colitis through ferroptotic mechanisms, whereas deferasirox reverses ferroptosis-related injury concurrently with histologic and microbiota-associated improvements [20,21]. Pathway-oriented studies have shown that ferroptosis can also be driven through epithelial signaling and iron-transport regulators, including STAT3, interferon regulatory factor 7 (IRF7)/microRNA-375-3p (miR-375-3p)/solute carrier family 11 member 2 (SLC11A2), and other pro-ferroptotic stress circuits, again with parallel changes in ROS, lipid peroxidation, iron accumulation, and epithelial injury [16,37]. More recent work has made the evidence even more complete by combining human tissue, DSS mice, cultured epithelial cells, total iron (Fe) and ferrous iron (Fe^2+^) quantification, oxidized lipid detection, transmission electron microscopy (TEM)-confirmed mitochondrial shrinkage and cristae loss, and partial rescue by ferrostatin-1 (Fer-1) or deferoxamine (DFO), which together provide one of the strongest multi-layered demonstrations of ferroptosis in colitis [19]. Additional intervention studies using chemically distinct agents have reproduced the same pattern of evidence, namely simultaneous improvement in disease phenotype and correction of ferroptosis readouts such as MDA, 4-hydroxynonenal (4-HNE), ACSL4, GPX4, iron accumulation, and mitochondrial injury [38,39]. The evidence that ferroptosis occurs in colitis is now supported by convergent findings from human tissues, animal models, epithelial cell systems, ultrastructural analyses, and rescue experiments. To assist researchers in navigating these varying experimental depths, Table 1 systematically categorizes these foundational breakthroughs across five distinct tiers of evidence, separating clinical human observations and classical animal systems from traditional cell lines, ex vivo organoids, and single-cell transcriptomics.

## 3. Cell-Type Specificity in Colitis-Associated Ferroptosis

Current evidence indicates that ferroptosis in colitis is strongly cell-context dependent, and the most firmly established ferroptotic compartment remains the intestinal epithelium. The earliest disease-specific study directly linked ferroptosis to intestinal epithelial cell death in UC, thereby placing epithelial ferroptosis at the center of mucosal injury rather than at the periphery of oxidative stress research [13]. Later work further consolidated this epithelial model by showing that ferroptosis in colon epithelial cells is itself a therapeutic target in UC [15]. Several mechanistic studies then localized ferroptotic regulation to epithelial signaling networks, including transporter- and kinase-linked control through SLC6A14/C/EBPβ-PAK6 and iron-transport regulation through IRF7/miR-375-3p/SLC11A2 [17,37]. Epithelial ferroptosis has also been linked to epithelial differentiation and mucosal repair capacity, because vitronectin-driven phosphodiesterase 4 (PDE4) activation was shown to impair intestinal epithelial cell (IEC) differentiation through ferroptosis, and PDE4 inhibition restored GPX4 and SLC7A11 together with mucosal healing indices [46]. Additional epithelial-focused studies have shown that epithelial ferroptotic vulnerability can be reduced by upregulating GPX4 and SLC7A11 or by modulating mechanosensitive signaling, further emphasizing that ferroptosis in colitis is not a generalized tissue event but a process tightly embedded in epithelial barrier biology [47,48]. Single-cell and epithelial-subset analyses have refined this view still further by showing that enterocyte-lineage cells are especially enriched for ferroptosis-associated signatures in UC, and that mitochondrial iron-redox control by GFER can restrain ferroptosis in colonic epithelial cells through poly(rC)-binding protein 1 (PCBP1)-mediated iron handling and peroxisome proliferator-activated receptor gamma coactivator 1-alpha (PGC-1α)/peroxisome proliferator-activated receptor gamma (PPARγ)-linked metabolic regulation [45]. Taken together, these studies support a clear conclusion that intestinal epithelial cells, especially enterocyte-associated epithelial populations, are currently the dominant and best-resolved ferroptotic compartment in colitis [15,45].

**Figure 1:**
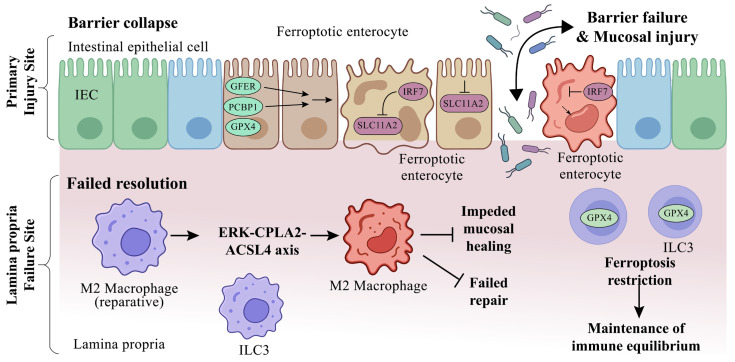
Cell-Type Specificity and Spatial Regulation of Ferroptosis. Epithelial ferroptosis, particularly in enterocytes, drives barrier collapse and mucosal injury through IRF7 and SLC11A2 signaling. In the lamina propria, ferroptosis of reparative M2 macrophages via the ERK-cPLA2-ACSL4 axis leads to resolution failure and failed repair, while GPX4 restricts ferroptosis in ILC3s to maintain immune equilibrium.

However, the field has moved beyond an epithelial-only model, and the most important conceptual shift has come from studies showing that ferroptosis in immune cells also shapes disease course, especially mucosal healing. The strongest evidence is the demonstration that M2 macrophages undergo ferroptosis in UC through the extracellular signal-regulated kinase (ERK)-cytosolic phospholipase A2 (cPLA2)-ACSL4 axis, and that this process impedes mucosal healing despite standard 5-aminosalicylic acid (5-ASA) treatment [23]. This finding is biologically important because it separates two pathogenic layers of ferroptosis in colitis: epithelial ferroptosis explains barrier collapse, whereas macrophage ferroptosis explains failed resolution and incomplete repair [23,49]. The broader macrophage literature is consistent with this interpretation, because studies of intestinal macrophages in IBD emphasize that M2-like macrophages are closely linked to tissue repair and homeostatic restoration, while alterations in the type 1 (M1)/M2 balance correlate with persistent inflammatory damage [50,51,52]. The immune-cell dimension extends beyond macrophages, because the GPX4-dependent restriction of ferroptosis in natural killer cell protein 46 positive (NKp46+) ILC3s was shown to be required for the control of intestinal inflammation, indicating that ferroptosis can influence innate lymphoid cell survival and immune equilibrium in the gut [24]. Systems-level analyses also support a broader immune interpretation by linking ferroptosis-related signatures in IBD to immune infiltration patterns and therapeutic response, thereby suggesting that ferroptosis in colitis should be understood as part of a multicellular immune microenvironment rather than as an epithelial event alone [28,53]. Overall, the present evidence supports a working hierarchy in which intestinal epithelial cells are the best-validated ferroptotic target, M2 macrophages are the strongest non-epithelial validated compartment, and additional immune populations such as ILC3s are emerging as important regulators of inflammation and healing in ferroptosis-associated colitis [25,54,55]. This working hierarchy of epithelial versus immune cell-specific ferroptosis is illustrated in Figure 1.

Despite the profound implications of these immune-cell findings, the concept of M2 macrophage ferroptosis must be interpreted with caution, as its specific contribution to colitis pathology remains in the early stages of validation. A primary limitation in the current literature is the heavy mechanistic reliance on a single major signaling model such as the classic ERK-cPLA2-ACSL4 cascade and a scarcity of cell-specific, in vivo genetic ablation systems [23]. Although independent translational validation has firmly established that macrophage dysregulation and shifts in M1/M2 polarization track closely with human IBD severity, direct, functional replication of macrophage-intrinsic ferroptotic execution during mucosal repair remains confined to a small number of seminal papers. Furthermore, future investigations must account for a sharp dichotomy in how ferroptosis operates across different chronological phases of colitis. During the acute inflammatory phase, ferroptosis behaves primarily as a destructive, cell-intrinsic oxidative event centered on the intestinal epithelium, where enterocyte loss directly drives barrier breakdown and precipitates the influx of luminal antigens. On the contrary, during the subsequent mucosal repair phase, the pathological impact of ferroptosis shifts from the epithelium to the immune microenvironment. In this resolution window, ferroptosis acts not as an initiator of injury, but as a barrier to recovery by selectively depleting the reparative M2 macrophage population required to coordinate tissue remodeling and tight-junction restoration. Recognizing this temporal and spatial heterogeneity is critical; blanket anti-ferroptotic interventions may yield highly divergent therapeutic outcomes depending on whether they are administered during an acute inflammatory flare or an active repair phase.

## 4. Mechanistic Networks Driving Ferroptosis in Colitis

Ferroptosis in colitis is not triggered by a single upstream lesion. It emerges when antioxidant defenses fail, membrane lipids become highly peroxidizable, and redox-active iron accumulates beyond the buffering capacity of the epithelium. The best-established execution module is the System Xc^−^/GSH/GPX4 axis, because repeated studies show that the suppression of SLC7A11, GSH depletion, and GPX4 loss are tightly linked to epithelial ferroptotic injury in colitis models [56,57,58]. This axis is further supported by studies showing that the reinforcement of GPX4-centered defense is sufficient to suppress ferroptosis and improve colitis severity, as observed with GPX4 induction, Nrf2/GPX4 activation, and kelch-like ECH-associated protein 1 (Keap1)/Nrf2/GPX4 pathway regulation [34,36,59]. The complementary pro-ferroptotic module is ACSL4-dependent phospholipid remodeling, because ACSL4 increases the pool of oxidizable membrane phospholipids and thereby amplifies lipid peroxidation once GPX4 protection declines [38,60,61]. Iron metabolism provides the third core layer, because iron overload, iron transport, and epithelial iron influx determine whether lipid peroxide stress progresses to ferroptotic execution. This principle is supported by work showing that excess iron aggravates colitis and by studies demonstrating that iron chelation reduces ferroptosis, inflammatory injury, and barrier dysfunction [20,21]. A more specific iron-transport mechanism was defined when IRF7 was shown to promote ferroptosis through the miR-375-3p/SLC11A2 axis, directly linking inflammatory signaling to epithelial iron uptake [37]. Metabolite-driven work has further extended this model by showing that bile acid-associated stress can activate HIF-2α/DMT1 signaling, increase ferrous iron accumulation, and intensify epithelial ferroptosis, thereby indicating that the iron module is dynamically coupled to the luminal metabolic environment [33]. Taken together, current evidence supports a core mechanistic architecture in which ferroptosis develops when the SLC7A11/GSH/GPX4 defense collapses, ACSL4-dependent lipid remodeling increases substrate vulnerability, and iron overload drives the propagation of lipid peroxidation [20,40,56].

**Figure 2:**
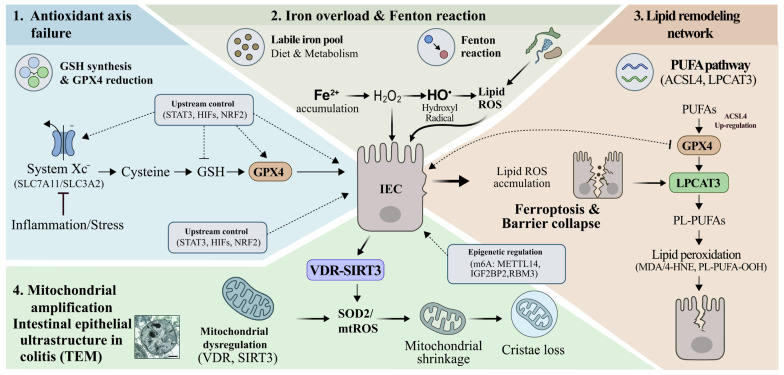
Mechanistic networks driving ferroptosis in colitis. This figure details the core biochemical execution modules and their upstream regulators in intestinal epithelial cells (IECs). The network consists of four primary nodes: (1) Antioxidant Axis Failure, centered on System Xc^−^, GSH synthesis, and GPX4; (2) Iron Overload and Fenton Reaction, involving diet and metabolic iron accumulation; (3) the Lipid Remodeling Network, driven by the polyunsaturated fatty acid (PUFA) pathway and ACSL4 upregulation; and (4) Mitochondrial Amplification, where dysregulation of the VDR-SIRT3-SOD2 axis leads to mtROS accumulation and characteristic cristae loss.

Upstream of this execution machinery, several signaling pathways actively reset the ferroptotic threshold in colitis rather than merely reflecting downstream injury. One important example is STAT3, because altered STAT3 signaling has been directly linked to ferroptosis in UC and appears to influence whether inflammatory stress is converted into epithelial death [16]. Hypoxia-adaptive signaling is also mechanistically relevant, as hypoxia-inducible factor-1 alpha (HIF-1α) has been shown to alleviate ferroptosis by preserving GPX4, indicating that the hypoxic mucosal environment can either worsen injury or buffer against ferroptotic execution depending on the balance of the pathway [61]. At the transcriptional and deubiquitination levels, BRCA1 associated protein 1 (BAP1) has emerged as another upstream regulator, because BAP1 upregulation in DSS colitis suppresses SLC7A11, increases oxidative stress and iron accumulation, and thereby promotes ferroptosis and bacterial translocation [62]. A distinct upstream network was defined by a CA9/STIM1 study, which connected ferroptosis to calcium homeostasis and lipid-synthesis regulators including insulin induced gene 2 (INSIG2), STIM1, sterol regulatory element-binding protein 1 (SREBP1), and stearoyl-CoA desaturase 1 (SCD1), thereby expanding the field beyond the classical GPX4–ACSL4 framework [18]. Mitochondrial amplification adds another major layer, because the VDR–SIRT3–SOD2–mtROS pathway controls mitochondrial ROS accumulation, oxidized lipid generation, and downstream ferroptotic injury in both the DSS-exposed intestine and epithelial cells [19]. This organelle-specific susceptibility is heavily governed by baseline metal-ion coordination and homeostatic maintenance. Recent comparative transcriptomic evidence across diverse mammalian tissues demonstrates that mitochondrial metal handling, particularly the precise regulation of iron transport, metal-binding architectures, and iron–sulfur (Fe-S) cluster assembly networks, is strictly required to maintain cellular redox equilibrium [63]. When these basal homeostatic pathways are disrupted, the accumulation of unbuffered mitochondrial iron not only accelerates local electron transport chain leakage and mtROS generation, but directly expands the labile iron pool, lowering the structural threshold for oxidative tissue injury and making the cell hypersensitive to ferroptotic execution cascades. Energy-sensing pathways also participate, as AMP-activated protein kinase (AMPK) activation suppresses ferroptosis and alleviates DSS colitis, indicating that metabolic-state correction can directly reshape ferroptotic susceptibility [64]. Additional work has shown that epithelial mechanosensing and differentiation programs intersect with ferroptosis, because Piezo-type mechanosensitive ion channel component 1 (Piezo1) deletion reduces barrier damage by regulating ferroptosis, whereas vitronectin/PDE4/protein kinase A (PKA)/cAMP response element-binding protein (CREB) signaling impairs epithelial differentiation through the ferroptosis-associated loss of GPX4 and SLC7A11 [40,41]. More recently, additional upstream regulators such as ACSF2, retinoid X receptor alpha (RXRA)/PPARG/PPARγ, and granzyme A (GZMA)–GPX4 have suggested that ferroptosis in colitis is also linked to fatty acid activation, nuclear-receptor signaling, and cytotoxic immune effector pathways, although these mechanisms remain less mature than the GPX4-, ACSL4-, or iron-centered modules [30,65,66]. Overall, the current literature supports the view that ferroptosis in colitis is a network-level threshold phenomenon in which inflammatory signaling, calcium and mitochondrial stress, iron handling, metabolic sensing, and epithelial differentiation programs converge on a limited set of execution nodes, especially SLC7A11, GPX4, ACSL4, and labile iron [26,55,67]. The integration of these execution modules and upstream regulators is mapped in Figure 2 and detailed in Table 2.

On the other hand, epithelial barrier failure in the colitic mucosa is rarely driven by a single cell death program acting in isolation. Emerging evidence indicates a high degree of biochemical crosstalk and spatial overlap between ferroptosis and other major forms of programmed cell death, including apoptosis, necroptosis, and pyroptosis. In the inflamed intestinal environment, a commitment of cell to a specific death pathway is highly fluid and governed by shared upstream triggers, particularly mitochondrial dysfunction and intracellular ROS amplification. For instance, the classic hallmarks of ferroptosis including lipid peroxidation and GSH depletion can directly impair mitochondrial outer membrane integrity, leading to cytochrome c release and the activation of cysteine-dependent aspartate-specific protease-3 (Caspase-3)-dependent intrinsic apoptosis. Parallel intersections exist with lytic death pathways. Pro-inflammatory cytokines, which are heavily enriched in the colitic microenvironment, simultaneously upregulate the pro-ferroptotic enzyme ACSL4 while stimulating the receptor-interacting protein kinase 1 (RIPK1)/receptor-interacting protein kinase 3 (RIPK3)/mixed lineage kinase domain-like protein (MLKL) necroptotic cascade. When execution takes place, the membrane permeabilization characteristic of necroptosis and gasdermin-D (GSDMD)-mediated pyroptosis alters intracellular ion gradients, triggering massive calcium influx via mechanosensitive or store-operated channels such as Piezo1 and STIM1. This intracellular calcium surge further accelerates cPLA2 activation and lipid peroxyl radical propagation, creating a positive-feedback loop that drives neighboring cells into ferroptosis. At the same time, the lytic rupture of cells undergoing ferroptosis or pyroptosis releases classic damage-associated molecular patterns (DAMPs) like high mobility group box 1 (HMGB1) into the lamina propria. These DAMPs bind toll-like receptor (TLR) on local immune cells, instigating an intense cytokine storm that feeds back onto healthy enterocytes, lower their threshold for all forms of cell death, and precipitates catastrophic barrier collapse. The molecular nodes bridging these pathways are systematically detailed in Table 3.

## 5. Epigenetic and Post-Transcriptional Regulation of Ferroptosis in Colitis

The epigenetic and post-transcriptional landscape of colitis-associated ferroptosis is now moving upstream from protein expression changes to RNA fate control, and the dominant evidence in this area currently centers on m6A-dependent regulation rather than on classical DNA methylation or histone modification. This distinction is important because UC itself already shows broad RNA-methylation abnormalities at the tissue level, including global m6A loss in colonic stem cells during severe disease, the existence of m6A-related molecular subtypes, and an altered RNA methylation machinery coupled with circulating m6A target genes that correlate with disease activity and inflammatory burden [70,71,72]. m6A regulation is also functionally relevant to colitis even before ferroptosis is specifically considered, because methyltransferase-like 3 (METTL3)-dependent RNA methylation has been shown to intensify colitic inflammation through ubiquitin-associated factor 1 (UAF1) stability and NLRP3 activation, YTH domain family 1 (YTHDF1)-dependent signaling has been linked to epithelial inflammatory amplification, and YTH domain containing 1 (YTHDC1)-dependent RNA regulation in gut macrophages contributes to intestinal homeostasis and barrier support [73,74,75]. The same principle extends to therapeutic immune regulation, because mesenchymal stem cell-derived exosomes can enhance M2 macrophage polarization through a METTL3–solute carrier family 37 member 2 (SLC37A2)–YTHDF1 post-transcriptional pathway, further indicating that RNA methylation is already embedded in the inflammatory and repair biology of IBD [76]. These studies collectively indicate that the colitic mucosa exists in an epitranscriptomically altered state, which creates the background from which ferroptosis-specific RNA regulation emerges.

**Figure 3:**
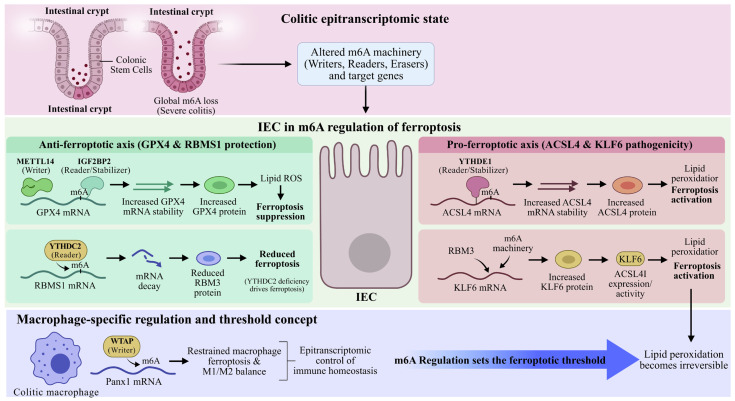
Epigenetic and post-transcriptional m6A regulation of ferroptosis. The diagram illustrates how the colitic epitranscriptomic state influences ferroptosis via RNA fate control. In the anti-ferroptotic axis, METTL14 and IGF2BP2 stabilize GPX4 mRNA to suppress ferroptosis, while YTHDC2 promotes RBMS1 decay. In the pro-ferroptotic axis, YTHDF1 and RBM3 stabilize ACSL4 and KLF6 mRNAs, respectively, to drive lipid peroxidation.

Direct ferroptosis-focused evidence now shows that post-transcriptional regulation in colitis acts on both the anti-ferroptotic arm and the pro-ferroptotic arm of the pathway. On the protective side, insulin-like growth factor 2 mRNA-binding protein 2 (IGF2BP2) suppresses epithelial ferroptosis by stabilizing m6A-modified GPX4 mRNA, thereby increasing GPX4 expression, lowering ROS, MDA, and iron accumulation, and improving DSS-induced disease severity [41]. Methyltransferase-like 14 (METTL14) appears to work at an even more upstream level by regulating the stability of m6A-modified GPX4 and thereby modulating colitis progression and ferroptosis [69]. On the pro-ferroptotic side, YTHDF1 promotes ferroptosis by stabilizing ACSL4 mRNA through m6A recognition, and its upregulation correlates with disease severity in both patient tissue and experimental colitis models [68]. This pro-ferroptotic direction has been expanded by newer work showing that RNA binding motif protein 3 (RBM3)-mediated m6A modification of Krüppel-like factor 6 (KLF6) promotes ACSL4-driven ferroptosis, thereby linking RNA-binding proteins, m6A control, transcriptional regulation, and lipid-remodeling machinery into one pathogenic axis [77]. Another emerging pathway shows that YTHDC2 deficiency stabilizes RNA binding motif single stranded interacting protein 1 (RBMS1) mRNA and drives epithelial ferroptosis, identifying a reader-dependent RNA-decay mechanism that restrains ferroptosis under normal conditions but is lost in colitis [42]. Post-transcriptional regulation is not confined to epithelial cells, because macrophage-associated work has shown that Wilms’ tumor 1-associating protein (WTAP)-dependent m6A modification of pannexin 1 (Panx1) restrains macrophage ferroptosis and polarization imbalance, suggesting that RNA methylation may also shape ferroptosis in the healing-associated immune compartment [78]. Taken together, these studies support a coherent model in which epitranscriptomic regulation controls ferroptosis in colitis by determining the stability or decay of a small number of pivotal transcripts, especially GPX4, ACSL4, RBMS1, KLF6, and Panx1. In practical terms, this means that RNA-level control does not merely fine-tune ferroptosis after it has begun. It helps set the ferroptotic threshold before lipid peroxidation becomes irreversible, which makes m6A readers, writers, and RNA-binding proteins attractive upstream targets for future therapies and biomarker development [79,80,81]. The m6A-RNA methylation axis and its control of pivotal transcripts are depicted in Figure 3.

## 6. Crosstalk with Microbiota, Metabolites, and Mucosal Healing

Ferroptosis in colitis cannot be interpreted only as a cell-intrinsic oxidative event, because the intestinal mucosa is continuously shaped by gut microbes, microbial metabolites, dietary substrates, and barrier-associated host responses. In this setting, several studies now indicate that microbiota remodeling and ferroptosis suppression often occur together rather than as unrelated parallel changes. Iron chelation with deferasirox reduces ferroptosis while simultaneously reshaping the intestinal microbiota and increasing short-chain fatty acid (SCFA) production, suggesting that iron availability, microbial ecology, and epithelial ferroptotic stress are tightly interconnected [21]. Butyrate provides the clearest example of a beneficial microbial metabolite acting directly on this axis, because it suppresses ferroptosis through Nrf2/GPX4-related signaling while improving intestinal barrier integrity [22]. Similar microbiota-linked protection has been reported for Buddlejasaponin IVb, which alleviates DSS colitis while inhibiting ferroptosis through the Nrf2/GPX4 pathway and correcting gut microbiota dysbiosis [82]. A related pattern was observed for Tremella aurantialba polysaccharides, which repaired the intestinal barrier, modulated gut microbiota composition and metabolites, and inhibited epithelial ferroptosis in DSS-induced colitis and RSL3-challenged epithelial cells [83]. These findings support a broader concept that the microbial environment may regulate ferroptotic threshold not only through inflammatory tone but also through postbiotic outputs, including amino acid and SCFA metabolism [83]. At the same time, this relationship is not uniformly protective. A high-fat dietary context can increase intestinal deoxycholic acid (DCA), which then promotes HIF-2α/DMT1-dependent iron loading and ferroptosis in intestinal epithelial cells, thereby exacerbating colonic inflammation [33]. This injurious side of the microbiota-metabolite axis is reinforced by work showing that dietary lipids can fuel GPX4-restricted enteritis, and that adherent-invasive *Escherichia coli* (AIEC), together with PUFAs, intensifies epithelial lipid peroxidation, GPX4 loss, and ferroptotic injury in IBD-related settings [84,85].

**Figure 4:**
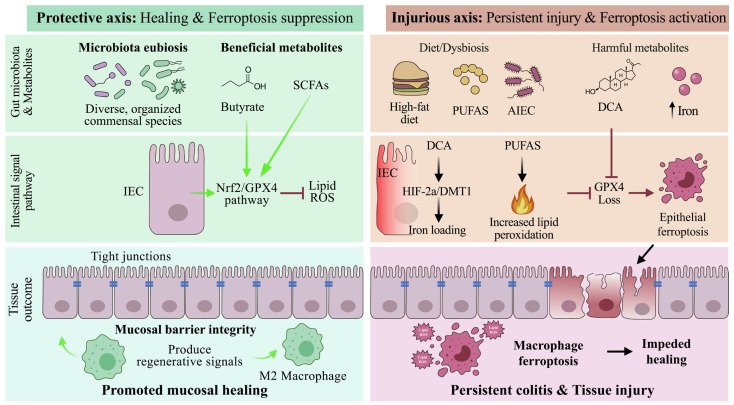
Crosstalk between environmental signals, ferroptosis, and healing. This integrated flowchart compares the protective and injurious axes shaping the colitic mucosa. The Protective Axis (**Left**) shows how microbiota eubiosis and beneficial metabolites like butyrate activate the Nrf2/GPX4 pathway to maintain mucosal barrier integrity and promote M2 macrophage-mediated healing. The Injurious Axis (**Right**) illustrates how diet-associated dysbiosis, harmful metabolites, and iron loading fuel epithelial and macrophage ferroptosis, leading to barrier failure, persistent colitis, and tissue injury. AIEC, Adherent-invasive *Escherichia coli*; DCA, Deoxycholic acid; IEC, Intestinal epithelial cell; PUFAs, Polyunsaturated fatty acids; ROS, Reactive oxygen species; SCFAs, Short-chain fatty acids.

The microbiota–ferroptosis connection is also important because it extends into the biology of mucosal healing rather than terminating at acute epithelial injury. One major advance was the demonstration that ferroptosis of M2 macrophages impedes mucosal healing in UC, indicating that ferroptosis can limit tissue recovery by depleting a reparative immune population rather than merely damaging epithelial cells [23]. This interpretation fits well with the broader concept that macrophage function is central to the transition from inflammation to repair in IBD, and that the perturbation of macrophage metabolism can prolong tissue injury and delay remission [49,86]. Barrier restoration itself is repeatedly linked to ferroptosis suppression. This is evident not only in butyrate-, deferasirox-, Buddlejasaponin IVb-, and Tremella-based studies, but also in work showing that GPX4-mediated ferroptosis control improves intestinal mucosal barrier function in IBD [21,22,66,82,83]. More broadly, the current literature suggests that ferroptosis is positioned at the interface of barrier failure, microbial dysbiosis, and repair-phase dysfunction, which explains why anti-ferroptotic benefits in colitis are often accompanied by an improved microbial balance, restored tight-junction proteins, and enhanced histologic healing rather than by the reduction in oxidative markers alone [53,87,88]. Taken together, these studies support a working model in which beneficial metabolites such as butyrate and microbiota-restoring interventions lower ferroptotic pressure and support healing, whereas harmful dietary and microbial signals such as deoxycholic acid, AIEC colonization, and PUFA-rich inflammatory contexts increase epithelial lipid peroxidation and shift the mucosa toward persistent injury [22,33,85]. The protective versus injurious axes of this environmental crosstalk are compared in Figure 4.

## 7. Therapeutic Strategies Targeting Ferroptosis in Colitis

Current therapeutic studies indicate that ferroptosis is not merely a mechanistic marker in colitis, but also a pharmacologically tractable vulnerability. The most direct proof-of-concept comes from classical ferroptosis inhibition, because Fer-1, Lip-1, deferiprone, and related anti-ferroptotic controls consistently attenuate DSS-induced injury concurrently with the correction of GPX4 loss, ACSL4 elevation, iron overload, lipid peroxidation, and histologic damage [14,89]. An iron-directed intervention provides especially strong mechanistic support, because deferasirox reduces ferrous iron accumulation, restores GPX4- and FTH-associated defense, improves disease activity, and simultaneously remodels the microbiota and SCFAoutputs [21]. Nutrient-based strategies are also becoming more persuasive, since butyrate suppresses ferroptosis while improving barrier integrity, selenium restores GPX4-centered defense in both patients and experimental systems, and vitamin D or 1,25-dihydroxyvitamin D3 (1,25(OH)_2_D_3_) restrains ferroptotic injury through ACSL4 suppression and the SIRT3–SOD2–mtROS pathway [19,22,40,90]. The therapeutic efficacy of these vitamin-dependent pathways relies on their fundamental role in stabilizing cellular architecture. Comprehensive tissue-wide transcriptomic profiling highlights that vitamin-dependent signaling networks and micronutrient-driven cofactors function as essential regulators of the mammalian metabolic state, cellular differentiation, and defenses against environmental stress. In detail, Vitamin D/VDR signaling directly modulates gene networks responsible for maintaining mitochondrial structural integrity and limiting membrane hyper-permeability. This broad biological foundation explains why localized vitamin supplementation and iron chelation are not merely symptomatic redox-scavenging interventions in colitis, but rather necessary corrections to fundamental mitochondrial and metabolic networks required to restore mucosal homeostasis [59]. A large body of work now shows that natural products and formula-based therapies repeatedly converge on the same anti-ferroptotic checkpoints, particularly Nrf2, SLC7A11, GPX4, ACSL4, and iron homeostasis. This is evident for curculigoside, Astragalus polysaccharide, Lizhong decoction, Shaoyao decoction, Shaoyao Gancao decoction, and Gancao Xiexin decoction, all of which alleviate colitis while suppressing ferroptotic hallmarks in intestinal tissue or epithelial cells [34,35,38,57,91,92]. Additional compounds further broaden this therapeutic spectrum, because β-caryophyllene, palmatine, celecoxib, α-lipoic acid, isorhamnetin, pinobanksin, biochanin A, and 5-O-methylvisammioside all show anti-colitic efficacy accompanied by the suppression of lipid peroxidation, the recovery of GPX4/SLC7A11 signaling, and the partial normalization of epithelial iron handling [39,58,93,94,95,96,97,98].

At the same time, the therapeutic literature is advancing from small molecules toward delivery-based strategies that attempt to improve specificity and biological context. Exosome studies are especially notable because human umbilical cord-mesenchymal stem cell (hUC-MSC)-derived vesicles carrying microRNA-129-5p (miR-129-5p) suppress ferroptosis through an ACSL4-dependent mechanism, while endometrial regenerative cell-derived exosomes reduce iron and MDA accumulation, increase GSH and GPX4 levels, decrease ACSL4 expression, and improve DSS-induced pathology [99,100]. Nanotechnology-based approaches extend this logic further, because targeted platforms designed to inhibit ferroptosis while promoting M2 macrophage polarization have shown therapeutic efficacy in experimental IBD, thereby suggesting that compartment-specific anti-ferroptotic delivery may be feasible [101]. Broader review analyses also emphasize that traditional Chinese medicine (TCM) and related phytochemical strategies may be particularly well suited to ferroptosis-targeted interventions because they often act on multiple nodes simultaneously, including iron handling, lipid peroxidation, antioxidant defenses, and mucosal repair [102,103,104]. However, these advances also reveal the main translational bottlenecks in the field. Most studies still rely on acute DSS models, many interventions combine anti-ferroptotic, anti-inflammatory, and barrier-protective effects that are difficult to rank causally, and only a minority include rigorous comparator designs or human validation [15,26,54]. Crucially, when evaluating the expansive therapeutic landscape of colitis-associated ferroptosis, researchers must distinguish between agents that directly intercept the cell death execution machinery and those that modulate ferroptosis indicators as a downstream consequence of broad antioxidant or anti-inflammatory effects. Because nutrients, traditional formulas, and natural products often possess multi-targeted properties, attributing their therapeutic success exclusively to ferroptosis blockade requires stringent experimental validation. To provide a clearer translational perspective, Table 4 systematically reorganizes current preclinical interventions into three functional tiers based on the stringency and directness of their anti-ferroptotic evidence, separating canonical execution inhibitors from multi-target contextual agents and emerging bioengineered delivery vehicles. The next step is to determine which of these strategies are best matched to epithelial injury, immune-cell repair failure, microbiota-associated dysregulation, and long-term mucosal healing in human disease [26,53,87].

## 8. Major Unresolved Problems and Future Directions

Despite rapid progress, the central challenge is no longer to show that ferroptosis exists in colitis, but to define when it is causal, in which cells it is dominant, and how it can be targeted safely in patients. Most current preclinical evidence relies on acute DSS injury models or bioinformatic predictions rather than on large patient cohorts with integrated molecular and clinical annotation. To bridge these gaps and transition from descriptive models to precision medicine, the field must actively address a series of interconnected pharmacological, diagnostic, and oncogenic bottlenecks.

### 8.1. Bioavailability and Delivery Constraints of Anti-Ferroptotic Interventions

The preclinical transition of the anti-ferroptotic strategies detailed in this review is heavily bottlenecked by distinct structural and physiological constraints. A primary hurdle applies to the extensive catalog of natural products and traditional phytochemical formulations. Despite displaying robust anti-ferroptotic capabilities in vitro, molecules such as curculigoside, astragalus polysaccharides, and various flavonoids frequently suffer from poor oral bioavailability, low aqueous solubility, and rapid first-pass hepatic metabolism. Maintaining therapeutic concentrations within the inflamed colonic epithelium via conventional oral administration remains exceedingly difficult, necessitating unphysiologically high doses that complicate clinical utility [43].

A distinct liability surrounds the systemic deployment of broad iron chelators. Although agents like deferasirox effectively deplete the mucosal labile iron pool to restrain lipid peroxidation, chronic or systemic administration poses a high risk of systemic iron deficiency, anemia, and off-target toxicities. Furthermore, systemic iron clearance may inadvertently restrict the iron required by non-pathogenic, beneficial commensal microbes, disrupting the very intestinal ecology these therapies aim to preserve.

To bypass these liabilities, bioengineered delivery platforms such as exosomes and targeted nanomedicines have emerged. However, the real-world feasibility of colon-targeted nanomedicines remains constrained by the highly altered physiology of the colitic gut, where accelerated transit time, massive diarrhea, and thick inflammatory exudate create physical barriers that hinder nanomaterials from adhering to or penetrating the epithelial mucosa. Furthermore, scaling up the manufacturing of biomimetic exosomes while preserving batch-to-batch reproducibility, structural stability in gastric fluid, and long-term storage viability presents an immense bioengineering hurdle.

### 8.2. Clinical Validation Pathways for Hub Gene Biomarkers

To transform bioinformatic hub genes such as LCN2, ACSF2, and MFN2 into clinically actionable instruments, future investigations must move beyond static bioinformatic predictions toward targeted, multi-cohort translational protocols [105]. A highly feasible approach is the implementation of Cross-Sectional Biopsy Profiling stratified by clinical phenotype. Mucosal punch biopsies collected during routine surveillance colonoscopies should be compared between active, treatment-refractory patients (e.g., those failing anti- tumor necrosis factor (TNF) or anti-Janus kinase (JAK) therapies), treatment-naive individuals, and patients in deep histologic remission. Quantitative polymerase chain reaction (PCR) and multiplex immunohistochemistry can then evaluate whether pro-ferroptotic drivers are selectively enriched in non-responding mucosal zones, and whether mitochondrial protective elements linearly track with mucosal healing indices.

Moving beyond tissue snapshots, a secondary priority must be Longitudinal Biofluid Monitoring. Because epithelial desquamation and barrier leakage dump intracellular contents into the bowel lumen, prospective clinical trials should track longitudinal expressions of these targets in fecal and serum samples collected at baseline, mid-induction, and post-maintenance therapy phases. Enzyme-linked immunosorbent assays (ELISA) validating secretory candidates like LCN2 can then be statistically correlated with fecal calprotectin levels, partial Mayo scores, and long-term relapse tracking to establish a clear path toward non-invasive treatment monitoring.

### 8.3. The Ferroptosis Paradox: Balancing Acute Cytoprotection with Onco-Surveillance

A critical, yet often overlooked, challenge in translating ferroptosis-targeted therapies is the double-edged function of iron-dependent cell death in the gut. Although inhibiting ferroptosis is clearly cytoprotective during acute flares of UC, the long-term consequences of such intervention must be weighed against the risk of colitis-associated cancer [44]. Ferroptosis serves as an evolutionary control mechanism to eliminate cells that have accumulated excessive oxidative damage or metabolic stress features of the dysplastic transition in a chronically inflamed colon.

This innate surveillance loop is directly hardwired into canonical tumor-suppressor networks. Most notably, the p53 pathway exerts a substantial portion of its anti-neoplastic surveillance by directly downregulating the transcription of SLC7A11, effectively sensitizing dysplastic clones to lipid peroxidation-mediated death before clonal expansion can take place. Similarly, the tumor suppressor BAP1 actively represses SLC7A11 to promote an environment sensitive to ferroptotic execution. Consequently, by pharmacologically reinforcing antioxidant defenses globally and chronically, clinicians may inadvertently create a pro-survival shield for precancerous clones that would otherwise be cleared by the host.

Reconciling this clinical paradox requires highly regulated kinetic and spatial gating. Anti-ferroptotic interventions must be deployed strictly as short-term induction agents during active disease flares to suppress hyper-acute enterocyte desquamation. Once clinical remission is established, the therapy must be cycled off to allow endogenous tumor-suppressor pathways to resume full functional surveillance. Balancing these immediate cytoprotective benefits against long-term oncogenic risks defines the next frontier of the field, establishing a demanding yet highly promising roadmap for true mechanism-based translation.

## 9. Conclusions

The synthesis of current literature establishes ferroptosis not as a passive, secondary byproduct of inflammation, but as a central, regulated driver of UC pathogenesis that offers unique opportunities for clinical intervention. As the field transitions from descriptive preclinical profiling to clinical application, the current state of colitis-associated ferroptosis research can be summarized across three clinical priorities:

(a) Most reliable current evidence: Convergent clinical and preclinical data prove that enterocyte ferroptosis drives oxidative mucosal barrier failure. This execution is consistently triggered by SLC7A11/GSH/GPX4 defense collapse, ACSL4-dependent lipid remodeling, and labile iron accumulation. Additionally, single-cell data confirm that ferroptosis occurs in reparative M2 macrophages, directly impeding mucosal resolution.

(b) Unresolved mechanisms: The exact threshold where dynamic epitranscriptomic RNA modifications transition into irreversible lipid peroxidation cascades remains unmapped. Crucially, because ferroptosis acts as an innate surveillance mechanism to eliminate highly damaged cells, the long-term cancer risk of systemic, chronic ferroptosis inhibition during the colitis-to-dysplasia transition remains a critical safety concern.

(c) Future translational directions: Biomarker development must transition toward validating multi-target panels in longitudinal clinical cohorts to predict endoscopic relapses. Concurrently, therapeutic engineering must prioritize compartment-specific, stimulus-responsive nanoplatforms or exosomes that selectively protect epithelial grids or M2 macrophages during active inflammatory flares without causing systemic iron toxicity or compromising long-term tumor surveillance.

## Figures and Tables

**Table 1:** Stratified evidence levels of original studies on ferroptosis in colitis.

Evidence Level/Model Category	Principal Cell Type/Compartment	Major Ferroptosis-Related Finding	Source
Tier I: Human Clinical & Translational Validation	Intestinal epithelial cells	Identified the presence and involvement of ferroptosis directly in human UC mucosal biopsy tissue.	[13]
	Colon epithelial cells	Positioned patient-derived colonic epithelial ferroptosis as a highly viable therapeutic target for UC.	[15]
	Patient colonic tissue	Demonstrated a severe pathogenetic reduction in mucosal VDR and SIRT3 expressions in clinical UC samples.	[19]
Tier II: In Vivo Animal Model Systems	Colonic epithelium/whole colon (DSS mice)	Showed that DSS induces a pro-ferroptotic change that can be reversed by canonical Fer-1 or Lip-1.	[14]
	Colonic tissue (DSS mice)	Demonstrated that iron chelation with Deferasirox reduces mucosal Fe^2+^, suppresses ferroptosis markers, and remodels microflora.	[21]
	DSS-induced colitis in mice	Validated that systemic Vitamin D administration attenuates disease severity by actively dampening ACSL4 expression.	[40]
Tier III: Traditional In Vitro Cell Line Platforms	Human Caco-2 adenocarcinoma cells	Established that Astragalus polysaccharide blocks RSL3- or erastin-induced ferroptotic injury via Nrf2/HO-1.	[35]
	DSS-treated human NCM460 cells	Proved that the m6A reader IGF2BP2 binds and directly stabilizes GPX4 mRNA to suppress lipid peroxidation.	[41]
	Challenged intestinal cell lines	Revealed that a severe genetic deficiency in YTHDC2 stabilizes RBMS1 transcripts to drive epithelial ferroptosis.	[42]
Tier IV: Advanced Physiologic Models	Mouse- and patient-derived organoids	Utilized complex 3D organoid architectures to demonstrate that Gegen Qinlian decoction limits epithelial cell death via GPX4 protection.	[43]
	Intestinal organoid systems	Proved that the Zhilining formula blocks ALOX15-mediated lipid peroxidation to preserve epithelial barrier continuity.	[44]
Tier V: High-Resolution Landscape	Single-cell transcriptomic analyses	Dissected multicellular signatures to prove enterocyte-lineage cells are heavily enriched for ferroptosis, gated by GFER-iron handling.	[45]

Abbreviations: 3D, Three-Dimensional; ACSL4, Acyl-CoA Synthetase Long Chain Family Member 4; ALOX15, Arachidonate 15-Lipoxygenase; Caco-2, Human Colon Adenocarcinoma Cell Line; DSS, Dextran Sulfate Sodium; Fe^2+^, Ferrous Iron; Fer-1, Ferrostatin-1; GFER, Growth Factor, Augmenter of Liver Regeneration; GPX4, Glutathione Peroxidase 4; HO-1, Heme Oxygenase-1; IGF2BP2, Insulin-Like Growth Factor 2 mRNA-Binding Protein 2; Lip-1, Liproxstatin-1; m6A, N6-Methyladenosine; mRNA, Messenger RNA; NCM460, Normal Colonic Mucosa 460 (Cell Line); Nrf2, Nuclear Factor Erythroid 2-Related Factor 2; RBMS1, RNA Binding Motif Single Stranded Interacting Protein 1; RSL3, RAS Selective Lethal 3; SIRT3, Sirtuin 3; UC, Ulcerative Colitis; VDR, Vitamin D Receptor; YTHDC2, YTH Domain Containing 2.

**Table 2:** Mechanistic pathways in colitis-associated ferroptosis.

Mechanistic Module	Core Ferroptotic Consequence in Colitis	Typical Evidence/Readouts in Colitis Studies	Representative Studies
Antioxidant defense failure	Weakens lipid peroxide detoxification and lowers the ferroptosis threshold in intestinal epithelial cells	GPX4↓, GSH↓, MDA/4-HNE↑, lipid ROS↑, worsened epithelial injury	[14,22,56,57]
Lipid remodeling and ferroptotic susceptibility	Increases incorporation of peroxidation-prone phospholipids and promotes membrane lipid damage	ACSL4↑, PTGS2/COX-2↑, oxidized lipids↑, epithelial injury↑	[18,38,40,68]
overload and iron transport dysregulation	Expands the labile iron pool and accelerates iron-dependent lipid peroxidation	Total iron↑, Fe^2+^↑, ROS↑, MDA↑, GPX4↓, FTH/TF abnormalities	[20,21,37]
Mitochondrial redox amplification	Amplifies oxidative injury and reinforces lipid peroxidation once antioxidant capacity declines	mtROS↑, SIRT3↓, Ac-SOD2↑, ACSL4↑, GPX4↓, shrunken mitochondria with reduced cristae	[19]
Inflammatory signaling control of ferroptotic sensitivity	Links cytokine/inflammatory signaling to ferroptotic commitment and epithelial injury	Altered STAT3 activity with parallel changes in ferroptosis markers and colitis severity	[16]
Transporter- and kinase-linked epithelial regulation	Couples nutrient transport and stress-responsive signaling to epithelial ferroptosis	Increased ferroptosis with pathway activation; reduced injury after pathway suppression	[17]
Epitranscriptomic and post-transcriptional control	Alters transcript stability of anti-ferroptotic or pro-ferroptotic genes, thereby shifting ferroptotic threshold upstream of protein execution	GPX4 mRNA stability↑ or ↓, ACSL4 mRNA stability↑, ROS/MDA/iron changes, colitis severity changes	[41,68,69]
Energy-sensing and metabolic restraint	Suppresses ferroptosis in DSS colitis, likely by improving stress adaptation and redox balance	Reduced ferroptosis-associated injury together with improved colitis phenotypes	[64]
Microbiota-metabolite crosstalk	Microbial ecology and metabolite output modify epithelial redox balance and ferroptosis burden	Butyrate-associated Nrf2/GPX4 support and barrier improvement; iron overload linked to ferroptosis and microbiota disruption	[21,22]
Emerging ferroptosis-related regulators not yet fully integrated	Suggests that additional lipid/iron-metabolic regulators may connect ferroptosis with immune pathways such as TLR and NF-κB signaling	ACSF2 downregulation in UC models; Fer-1 reversibility in cell models; association with immune-related pathways	[30]

Abbreviations: 4-HNE, 4-Hydroxynonenal; ACSF2, Acyl-CoA Synthetase Family Member 2; ACSL4, Acyl-CoA Synthetase Long Chain Family Member 4; Ac-SOD2, Acetylated Superoxide Dismutase 2; COX-2, Cyclooxygenase-2; DSS, Dextran Sulfate Sodium; Fe^2+^, Ferrous Iron; Fer-1, Ferrostatin-1; FTH, Ferritin Heavy Chain; GPX4, Glutathione Peroxidase 4; GSH, Glutathione; MDA, Malondialdehyde; mRNA, Messenger RNA; mtROS, Mitochondrial Reactive Oxygen Species; NF-κB, Nuclear Factor-Kappa B; Nrf2, Nuclear Factor Erythroid 2-Related Factor 2; PTGS2, Prostaglandin-Endoperoxide Synthase 2; ROS, Reactive Oxygen Species; SIRT3, Sirtuin 3; STAT3, Signal Transducer and Activator of Transcription 3; TF, Transferrin; TLR, Toll-Like Receptor; UC, Ulcerative Colitis.

**Table 3:** Crosstalk between ferroptosis and co-existing cell death pathways in colitis.

Co-Existing Death Pathway	Primary Shared Nodes	Mechanistic Bridge to Ferroptosis	Impact on Mucosal Barrier Collapse
Apoptosis	Mitochondrial ROS, Cytochrome c, Caspase-3, tumor protein 53 (p53) signaling.	GPX4 depletion and lipid ROS distort mitochondrial membranes, inducing cytochrome c leakage to trigger apoptosis. Conversely, p53 activation can suppress SLC7A11 transcription, directly lowering the ferroptotic threshold.	Initiates orderly enterocyte extrusion; however, extensive concurrent apoptosis and ferroptosis overwhelm epithelial renewal capacity, leading to focal denudation of the mucosa.
Necroptosis	TNF-α, RIPK1, RIPK3, MLKL membrane pore formation.	TNF-α signaling simultaneously induces MLKL phosphorylation and upregulates ACSL4 expression. MLKL-driven membrane porous breakdown disrupts ionic homeostasis, accelerating calcium-dependent lipid peroxidation.	Triggers lytic epithelial desquamation and rapid paracellular permeability, opening structural gaps for luminal bacterial translocation.
Pyroptosis	NOD-like receptor family pyrin domain containing 3 (NLRP3) inflammasome, Caspase-1, GSDMD, HMGB1 release.	Ferroptotic lipid peroxides act as intrinsic danger signals that activate the NLRP3 inflammasome. Meanwhile, GSDMD pores induce calcium influx that drives cPLA2-dependent lipid remodeling, amplifying ferroptosis susceptibility.	Massive release of intracellular contents and inflammatory DAMPs into the lamina propria, inducing a feed-forward cytokine storm that destroys the tight-junction matrix.

Abbreviations: ACSL4, Acyl-CoA Synthetase Long Chain Family Member 4; Caspase-1, Cysteine-Dependent Aspartate-Specific Protease-1; Caspase-3, Cysteine-Dependent Aspartate-Specific Protease-3; cPLA2, Cytosolic Phospholipase A2; DAMPs, Damage-Associated Molecular Patterns; GPX4, Glutathione Peroxidase 4; GSDMD, Gasdermin D; HMGB1, High Mobility Group Box 1; MLKL, Mixed Lineage Kinase Domain-Like Protein; NLRP3, NOD-Like Receptor Family Pyrin Domain Containing 3; p53, Tumor Protein 53; RIPK1, Receptor-Interacting Protein Kinase 1; RIPK3, Receptor-Interacting Protein Kinase 3; ROS, Reactive Oxygen Species; SLC7A11, Solute Carrier Family 7 Member 11; TNF-α, Tumor Necrosis Factor Alpha.

**Table 4:** Classification of anti-ferroptotic interventions by experimental evidence level.

Evidence Classification	Representative Intervention	Primary Anti-Ferroptotic Axis/Targeted Node	Representative Source
Class A: Direct/Canonical Rescue Evidence	Fer-1/Lip-1	Direct lipid peroxyl radical scavenging; complete block of membrane lipid peroxidation and GPX4 destruction.	[14,89]
	Deferasirox	Depletion of the mucosal labile iron pool; direct blockade of Fenton reaction propagation.	[21]
	Butyrate	Direct activation of the Nrf2/GPX4 defense pathway to block lipid peroxidation.	[22]
	Vitamin D/1,25(OH)_2_D_3_	Direct transcriptional suppression of ACSL4 expression and protection of the SIRT3-SOD2 mitochondrial core.	[19,40]
	Selenium	Obligate cofactor delivery to directly induce intracellular GPX4 synthesis and antioxidant gating.	[90]
Class B: Multi-Target Contextual Evidence	Astragalus polysaccharide	Up-regulation of Nrf2/HO-1 signaling cascades to decrease oxidative cell loss.	[35]
	Lizhong decoction	Protection of enterocytes via the broader Nrf2/SLC7A11/GPX4 pathway.	[57]
	Buddlejasaponin IVb	Coordinated Nrf2/GPX4 pathway upregulation and correction of gut microbial dysbiosis.	[82]
	Gancao Xiexin decoction	Associated suppression of inflammatory cytokine-driven ACSL4 upregulation.	[38]
	Palmatine/Isorhamnetin	Concurrent iron chelation and Nrf2/HO-1 antioxidant loop activation.	[93,96]
Class C: Emerging Bioengineered Platforms	hUC-MSC Exosomes (miR-129-5p)	Targeted delivery of regulatory RNA to selectively post-transcriptionally suppress ACSL4 expression.	[99]
	Endometrial Regenerative Cell Exosomes	Downregulation of intestine ferroptosis markers.	[100]
	M2 Polarization Nanoparticles	Coordinated biomimetic delivery to inhibit epithelial ferroptosis while promoting M2 macrophage tissue repair.	[101]

Abbreviations: 1,25(OH)_2_D_3_, 1,25-Dihydroxyvitamin D3; ACSL4, Acyl-CoA Synthetase Long Chain Family Member 4; Fer-1, Ferrostatin-1; GPX4, Glutathione Peroxidase 4; HO-1, Heme Oxygenase-1; hUC-MSC, Human Umbilical Cord-Mesenchymal Stem Cell; Lip-1, Liproxstatin-1; M2, Type 2 Macrophage (Alternatively Activated Macrophage); miR-129-5p, microRNA-129-5p; Nrf2, Nuclear Factor Erythroid 2-Related Factor 2; RNA, Ribonucleic Acid; SIRT3, Sirtuin 3; SLC7A11, Solute Carrier Family 7 Member 11; SOD2, Superoxide Dismutase 2.

## Data Availability

No new data were created or analyzed in this study.

## Abbreviations

The following abbreviations are used in this manuscript:

1,25(OH)_2_D_3_	1,25-Dihydroxyvitamin D3
4-HNE	4-Hydroxynonenal
5-ASA	5-Aminosalicylic Acid
Ac-SOD2	Acetylated Superoxide Dismutase 2
ACSF2	Acyl-CoA Synthetase Family Member 2
ACSL4	Acyl-CoA Synthetase Long Chain Family Member 4
AIEC	Adherent-Invasive **Escherichia coli**
ALOX15	Arachidonate 15-Lipoxygenase
AMPK	AMP-Activated Protein Kinase
BAP1	BRCA1 Associated Protein 1
CA9	Carbonic Anhydrase 9
Caco-2	Human Colon Adenocarcinoma Cell Line
CBS	Cystathionine Beta-Synthase
COX-2	Cyclooxygenase-2
cPLA2	Cytosolic Phospholipase A2
CREB	cAMP Response Element-Binding Protein
C/EBPβ	CCAAT/Enhancer-Binding Protein Beta
DAMPs	Damage-Associated Molecular Patterns
DCA	Deoxycholic Acid
DFO	Deferoxamine
DMT1	Divalent Metal Transporter 1
DPP4	Dipeptidyl Peptidase 4
DSS	Dextran Sulfate Sodium
ELISA	Enzyme-Linked Immunosorbent Assay
ERK	Extracellular Signal-Regulated Kinase
Fe	Iron
Fe-S	Iron–Sulfur
Fe^2+^	Ferrous Iron
Fer-1	Ferrostatin-1
FTH1	Ferritin Heavy Chain 1
GFER	Growth Factor, Augmenter of Liver Regeneration (Erv1-like)
GPX4	Glutathione Peroxidase 4
GSDMD	Gasdermin D
GSH	Glutathione
GZMA	Granzyme A
HIF-1α	Hypoxia-Inducible Factor-1 Alpha
HIF-2α	Hypoxia-Inducible Factor-2 Alpha
HMGB1	High Mobility Group Box 1
HO-1	Heme Oxygenase-1
hUC-MSC	Human Umbilical Cord-Mesenchymal Stem Cell
IBD	Inflammatory Bowel Disease
IEC	Intestinal Epithelial Cell
IGF2BP2	Insulin-Like Growth Factor 2 mRNA-Binding Protein 2
ILC3s	Group 3 Innate Lymphoid Cells
INSIG2	Insulin Induced Gene 2
IRF7	Interferon Regulatory Factor 7
JAK	Janus Kinase
Keap1	Kelch-Like ECH-Associated Protein 1
KLF6	Krüppel-Like Factor 6
LCN2	Lipocalin 2
Lip-1	Liproxstatin-1
M1	Type 1 Macrophage (Classically Activated Macrophage)
M2	Type 2 Macrophage (Alternatively Activated Macrophage)
m6A	N6-Methyladenosine
MDA	Malondialdehyde
METTL3	Methyltransferase-Like 3
METTL14	Methyltransferase-Like 14
MFN2	Mitofusin 2
miR-129-5p	microRNA-129-5p
miR-375-3p	microRNA-375-3p
MLKL	Mixed Lineage Kinase Domain-Like Protein
mRNA	Messenger RNA
mtROS	Mitochondrial Reactive Oxygen Species
NCM460	Normal Colonic Mucosa 460 (Cell Line)
NF-κB	Nuclear Factor-Kappa B
NKp46^+^	Natural Killer Cell Protein 46 Positive
NLRP3	NOD-Like Receptor Family Pyrin Domain Containing 3
Nrf2	Nuclear Factor Erythroid 2-Related Factor 2
p53	Tumor Protein 53
PAK6	p21-Activated Kinase 6
Panx1	Pannexin 1
PCBP1	Poly(rC)-Binding Protein 1
PCR	Polymerase Chain Reaction
PDE4	Phosphodiesterase 4
PGC-1α	Peroxisome Proliferator-Activated Receptor Gamma Coactivator 1-Alpha
Piezo1	Piezo-type Mechanosensitive Ion Channel Component 1
PKA	Protein Kinase A
PPARG/PPARγ	Peroxisome Proliferator-Activated Receptor Gamma
PTGS2	Prostaglandin-Endoperoxide Synthase 2
PUFA	Polyunsaturated Fatty Acid
RBM3	RNA Binding Motif Protein 3
RBMS1	RNA Binding Motif Single Stranded Interacting Protein 1
RIPK1	Receptor-Interacting Protein Kinase 1
RIPK3	Receptor-Interacting Protein Kinase 3
RNA	Ribonucleic Acid
ROS	Reactive Oxygen Species
RSL3	RAS Selective Lethal 3
RXRA	Retinoid X Receptor Alpha
SCD1	Stearoyl-CoA Desaturase 1
SCFA	Short-Chain Fatty Acid
SIRT3	Sirtuin 3
SLC6A14	Solute Carrier Family 6 Member 14
SLC7A11	Solute Carrier Family 7 Member 11
SLC11A2	Solute Carrier Family 11 Member 2
SLC37A2	Solute Carrier Family 37 Member 2
SOD2	Superoxide Dismutase 2
SREBP1	Sterol Regulatory Element-Binding Protein 1
STAT3	Signal Transducer and Activator of Transcription 3
STIM1	Stromal Interaction Molecule 1
System Xc^−^	Cystine/Glutamate Antiporter
TCM	Traditional Chinese Medicine
TEM	Transmission Electron Microscopy
TF	Transferrin
TLR	Toll-Like Receptor
TNF-α	Tumor Necrosis Factor Alpha
UAF1	Ubiquitin-Associated Factor 1
UC	Ulcerative Colitis
VDR	Vitamin D Receptor
WTAP	Wilms’ Tumor 1-Associating Protein
YTHDC1	YTH Domain Containing 1
YTHDC2	YTH Domain Containing 2
YTHDF1	YTH Domain Family 1

## References

[1] Ramos G.P., Papadakis K.A. (2019). Mechanisms of Disease: Inflammatory Bowel Diseases. Mayo Clin. Proc..

[2] Wu J., Xu X., Duan J., Chai Y., Song J., Gong D., Wang B., Hu Y., Han T., Ding Y. (2024). EFHD2 suppresses intestinal inflammation by blocking intestinal epithelial cell TNFR1 internalization and cell death. Nat. Commun..

[3] Rana N., Privitera G., Kondolf H.C., Bulek K., Lechuga S., De Salvo C., Corridoni D., Antanaviciute A., Maywald R.L., Hurtado A.M. (2022). GSDMB is increased in IBD and regulates epithelial restitution/repair independent of pyroptosis. Cell.

[4] Dixon S.J., Lemberg K.M., Lamprecht M.R., Skouta R., Zaitsev E.M., Gleason C.E., Patel D.N., Bauer A.J., Cantley A.M., Yang W.S. (2012). Ferroptosis: An iron-dependent form of nonapoptotic cell death. Cell.

[5] Tang D., Chen X., Kang R., Kroemer G. (2021). Ferroptosis: Molecular mechanisms and health implications. Cell Res..

[6] Stockwell B.R. (2022). Ferroptosis turns 10: Emerging mechanisms, physiological functions, and therapeutic applications. Cell.

[7] Jiang X., Stockwell B.R., Conrad M. (2021). Ferroptosis: Mechanisms, biology and role in disease. Nat. Rev. Mol. Cell Biol..

[8] Sun Y., Chen P., Zhai B., Zhang M., Xiang Y., Fang J., Xu S., Gao Y., Chen X., Sui X. (2020). The emerging role of ferroptosis in inflammation. Biomed. Pharmacother..

[9] Ru Q., Li Y., Chen L., Wu Y., Min J., Wang F. (2024). Iron homeostasis and ferroptosis in human diseases: Mechanisms and therapeutic prospects. Signal Transduct. Target. Ther..

[10] Ursini F., Maiorino M. (2020). Lipid peroxidation and ferroptosis: The role of GSH and GPx4. Free Radic. Biol. Med..

[11] Stockwell B.R., Friedmann Angeli J.P., Bayir H., Bush A.I., Conrad M., Dixon S.J., Fulda S., Gascon S., Hatzios S.K., Kagan V.E. (2017). Ferroptosis: A Regulated Cell Death Nexus Linking Metabolism, Redox Biology, and Disease. Cell.

[12] Berndt C., Alborzinia H., Amen V.S., Ayton S., Barayeu U., Bartelt A., Bayir H., Bebber C.M., Birsoy K., Bottcher J.P. (2024). Ferroptosis in health and disease. Redox Biol..

[13] Xu M., Tao J., Yang Y., Tan S., Liu H., Jiang J., Zheng F., Wu B. (2020). Ferroptosis involves in intestinal epithelial cell death in ulcerative colitis. Cell Death Dis..

[14] Chen Y., Zhang P., Chen W., Chen G. (2020). Ferroptosis mediated DSS-induced ulcerative colitis associated with Nrf2/HO-1 signaling pathway. Immunol. Lett..

[15] Yokote A., Imazu N., Umeno J., Kawasaki K., Fujioka S., Fuyuno Y., Matsuno Y., Moriyama T., Miyawaki K., Akashi K. (2023). Ferroptosis in the colon epithelial cells as a therapeutic target for ulcerative colitis. J. Gastroenterol..

[16] Huang F., Zhang S., Li X., Huang Y., He S., Luo L. (2022). STAT3-mediated ferroptosis is involved in ulcerative colitis. Free Radic. Biol. Med..

[17] Chen Y., Yan W., Chen Y., Zhu J., Wang J., Jin H., Wu H., Zhang G., Zhan S., Xi Q. (2022). SLC6A14 facilitates epithelial cell ferroptosis via the C/EBPβ-PAK6 axis in ulcerative colitis. Cell. Mol. Life Sci..

[18] Ni J., Zhang L., Feng G., Bao W., Wang Y., Huang Y., Chen T., Chen J., Cao X., You K. (2024). Vanillic acid restores homeostasis of intestinal epithelium in colitis through inhibiting CA9/STIM1-mediated ferroptosis. Pharmacol. Res..

[19] Wang H.Q., Zhu Y.W., Dou Z.Y., Chen Z., Tong C.C., He X., Ma X.H., Guan J., Xu D.X., Chen X. (2025). 1,25(OH)_2_D_3_ ameliorates DSS-induced intestinal ferroptosis through the SIRT3-SOD2-mtROS pathway. J. Nutr. Biochem..

[20] Gu K., Wu A., Yu B., Zhang T., Lai X., Chen J., Yan H., Zheng P., Luo Y., Luo J. (2023). Iron overload induces colitis by modulating ferroptosis and interfering gut microbiota in mice. Sci. Total Environ..

[21] Wu Y., Ran L., Yang Y., Gao X., Peng M., Liu S., Sun L., Wan J., Wang Y., Yang K. (2023). Deferasirox alleviates DSS-induced ulcerative colitis in mice by inhibiting ferroptosis and improving intestinal microbiota. Life Sci..

[22] Chen H., Qian Y., Jiang C., Tang L., Yu J., Zhang L., Dai Y., Jiang G. (2024). Butyrate ameliorated ferroptosis in ulcerative colitis through modulating Nrf2/GPX4 signal pathway and improving intestinal barrier. Biochim. Biophys. Acta Mol. Basis Dis..

[23] Ye Y., Liu L., Feng Z., Liu Y., Miao J., Wei X., Li H., Yang J., Cao X., Zhao J. (2024). The ERK-cPLA2-ACSL4 axis mediating M2 macrophages ferroptosis impedes mucosal healing in ulcerative colitis. Free Radic. Biol. Med..

[24] Li X., He J., Gao X., Zheng G., Chen C., Chen Y., Xing Z., Wang T., Tang J., Guo Y. (2024). GPX4 restricts ferroptosis of NKp46^+^ILC3s to control intestinal inflammation. Cell Death Dis..

[25] Zhang X., Ma Y., Lv G., Wang H. (2023). Ferroptosis as a therapeutic target for inflammation-related intestinal diseases. Front. Pharmacol..

[26] Long D., Mao C., Huang Y., Xu Y., Zhu Y. (2024). Ferroptosis in ulcerative colitis: Potential mechanisms and promising therapeutic targets. Biomed. Pharmacother..

[27] Kao A.T., Cabanlong C.V., Padilla K., Xue X. (2024). Unveiling ferroptosis as a promising therapeutic avenue for colorectal cancer and colitis treatment. Acta Pharm. Sin. B.

[28] Tang H., Li P., Guo X. (2023). Ferroptosis-Mediated Immune Microenvironment and Therapeutic Response in Inflammatory Bowel Disease. DNA Cell Biol..

[29] Deng L., He S., Li Y., Ding R., Li X., Guo N., Luo L. (2023). Identification of Lipocalin 2 as a Potential Ferroptosis-related Gene in Ulcerative Colitis. Inflamm. Bowel Dis..

[30] Luo L., Zhang S., Guo N., Li H., He S. (2023). ACSF2-mediated ferroptosis is involved in ulcerative colitis. Life Sci..

[31] Zhai L., Pan H., Guo Z., Zhou W., Ding Q., Wang H., Chen Q., Yao P. (2025). Molecular mechanisms of ferroptosis in ulcerative colitis: Insights from machine learning, WGCNA, and immune cell infiltration analysis. Front. Immunol..

[32] Qian R., Tang M., Ouyang Z., Cheng H., Xing S. (2023). Identification of ferroptosis-related genes in ulcerative colitis: A diagnostic model with machine learning. Ann. Transl. Med..

[33] Wang C., Chu Q., Dong W., Wang X., Zhao W., Dai X., Liu W., Wang B., Liu T., Zhong W. (2024). Microbial metabolite deoxycholic acid-mediated ferroptosis exacerbates high-fat diet-induced colonic inflammation. Mol. Metab..

[34] Wang S., Liu W., Wang J., Bai X. (2020). Curculigoside inhibits ferroptosis in ulcerative colitis through the induction of GPX4. Life Sci..

[35] Chen Y., Wang J., Li J., Zhu J., Wang R., Xi Q., Wu H., Shi T., Chen W. (2021). Astragalus polysaccharide prevents ferroptosis in a murine model of experimental colitis and human Caco-2 cells via inhibiting NRF2/HO-1 pathway. Eur. J. Pharmacol..

[36] Dong S., Lu Y., Peng G., Li J., Li W., Li M., Wang H., Liu L., Zhao Q. (2021). Furin inhibits epithelial cell injury and alleviates experimental colitis by activating the Nrf2-Gpx4 signaling pathway. Dig. Liver Dis..

[37] Chen Z., Gu Q., Chen R. (2023). Promotive role of IRF7 in ferroptosis of colonic epithelial cells in ulcerative colitis by the miR-375-3p/SLC11A2 axis. Biomol. Biomed..

[38] Pan Z., Gan C., Zhi S., Yang Y., Zhang Y., Li L., Zhang S., Huang Q. (2025). Gancao Xiexin decoction attenuated experimental colitis through suppressing ACSL4-mediated ferroptosis. J. Ethnopharmacol..

[39] Wu Y.T., Zhong L.S., Huang C., Guo Y.Y., Jin F.J., Hu Y.Z., Zhao Z.B., Ren Z., Wang Y.F. (2022). β-Caryophyllene Acts as a Ferroptosis Inhibitor to Ameliorate Experimental Colitis. Int. J. Mol. Sci..

[40] Gao S., Sun C., Kong J. (2023). Vitamin D Attenuates Ulcerative Colitis by Inhibiting ACSL4-Mediated Ferroptosis. Nutrients.

[41] Liu W., Zeng H. (2024). IGF2BP2 attenuates intestinal epithelial cell ferroptosis in colitis by stabilizing m^6^A-modified GPX4 mRNA. Cytokine.

[42] Qiu B., Fu Z., Wang H., Bai F. (2026). YTHDC2 Deficiency Exacerbates Ulcerative Colitis by Stabilizing RBMS1 mRNA to Drive Epithelial Ferroptosis. Inflammation.

[43] Zheng Y., Yan F., He S., Luo L. (2024). Targeting ferroptosis in autoimmune diseases: Mechanisms and therapeutic prospects. Autoimmun. Rev..

[44] Zhou R., Dai P., Li J., Guo C., Wang M., Liu F., Lin C., Zhu C. (2025). Zhilining formula suppresses ferroptosis in colonic epithelial cells by inhibiting ALOX15/15(S)-HPETE to repress colorectal tumorigenesis and progression. Phytomedicine.

[45] Song Y., Tan F., Song Q., Liao X., Mei Z., Lv L. (2025). Single-Cell Transcriptomics Unravels Growth Factor Erv1-Like Mediated Ferroptosis as a Key Driver of Intestinal Epithelial Dysfunction in Ulcerative Colitis. Adv. Sci..

[46] Pan W., Xiang L., Liang X., Du W., Zhao J., Zhang S., Zhou X., Geng L., Gong S., Xu W. (2023). Vitronectin Destroyed Intestinal Epithelial Cell Differentiation through Activation of PDE4-Mediated Ferroptosis in Inflammatory Bowel Disease. Mediat. Inflamm..

[47] Zhu J., Wu Y., Zhang L., Bai B., Han W., Wang H., Mei Q. (2024). Epithelial Piezo1 deletion ameliorates intestinal barrier damage by regulating ferroptosis in ulcerative colitis. Free Radic. Biol. Med..

[48] Wang H., Sun Y., Zhang X.X., Wang X.Y., Xia Y.J., Wang L.S. (2023). Tanshinone IIA protects intestinal epithelial cells from ferroptosis through the upregulation of GPX4 and SLC7A11. Biocell.

[49] Na Y.R., Stakenborg M., Seok S.H., Matteoli G. (2019). Macrophages in intestinal inflammation and resolution: A potential therapeutic target in IBD. Nat. Rev. Gastroenterol. Hepatol..

[50] Wright P.B., McDonald E., Bravo-Blas A., Baer H.M., Heawood A., Bain C.C., Mowat A.M., Clay S.L., Robertson E.V., Morton F. (2021). The mannose receptor (CD206) identifies a population of colonic macrophages in health and inflammatory bowel disease. Sci. Rep..

[51] Lu H., Suo Z., Lin J., Cong Y., Liu Z. (2024). Monocyte-macrophages modulate intestinal homeostasis in inflammatory bowel disease. Biomark. Res..

[52] Zhang M., Li X., Zhang Q., Yang J., Liu G. (2023). Roles of macrophages on ulcerative colitis and colitis-associated colorectal cancer. Front. Immunol..

[53] Xie H., Cao C., Shu D., Liu T., Zhang T. (2024). The important role of ferroptosis in inflammatory bowel disease. Front. Med..

[54] Ye Y., Liu L., Jing Y., Yao S., Yang M., Dai X., Piao M., Xu X., Feng Z., Wang X. (2024). Ferroptosis: A therapeutic opportunity of inflammatory bowel disease. Chin. Med. J..

[55] Ma B., Hu X., Ai X., Zhang Y. (2024). Research progress of ferroptosis and inflammatory bowel disease. Biometals.

[56] Zheng S., Yin J., Wang B., Ye Q., Huang J., Liang X., Wu J., Yue H., Zhang T. (2025). Polydatin protects against DSS-induced ulcerative colitis via Nrf2/Slc7a11/Gpx4-dependent inhibition of ferroptosis signalling activation. Front. Pharmacol..

[57] Li W., Wang Y., Zhang Y., Fan Y., Liu J., Zhu K., Jiang S., Duan J. (2024). Lizhong decoction ameliorates ulcerative colitis by inhibiting ferroptosis of enterocytes via the Nrf2/SLC7A11/GPX4 pathway. J. Ethnopharmacol..

[58] Bi H., Guo S., Wang Y., Liu Z., Wu G., Huo X., Guo L., Guo H., Xiong Y. (2024). Pinobanksin ameliorated DSS-induced acute colitis mainly through modulation of SLC7A11/glutathione-mediated intestinal epithelial ferroptosis. Food Funct..

[59] Wang J., Chen Q., Chu S., Zhu F., Zhang L., Yi Z., Li J., Hu D., Fan H., Yu T. (2025). Compound Sophorae Decoction Alleviates Ferroptosis in Colitis Rats via Activating Keap1/Nrf2/GPX4 Signaling Pathway. Gastroenterol. Res. Pract..

[60] Lam I.H., Chan C.I., Han M., Li L., Yu H.H. (2024). ACSL4 mediates inflammatory bowel disease and contributes to LPS-induced intestinal epithelial cell dysfunction by activating ferroptosis and inflammation. Open Med..

[61] Hu W., Cai Y., Cai D., Chen Z., Huang S., Zhang S., Zhuang H., Fang T., Chen X. (2025). HIF-1α alleviates ferroptosis in ulcerative colitis by regulation of GPX4. Cell Death Dis..

[62] Chen X., Yang Y., Han B., Zhou Z., Xu C., Gu M., Huang Z., Liu H., Ren K., Luan Y. (2025). BAP1 exacerbates inflammatory bowel disease by promoting ferroptosis via SLC7A11 suppression. Int. Immunopharmacol..

[63] Sadeesh E.M., Lahamge M.S., Singh P., Mohiddin R. (2025). Tissue-Specific Transcriptomic Profiling of Vitamin-Dependent Mitochondrial Pathways in Female Buffalo. Cell Biochem. Biophys..

[64] Sun S.P., Lu Y.F., Li H., Weng C.Y., Chen J.J., Lou Y.J., Lyu D., Lyu B. (2023). AMPK activation alleviated dextran sulfate sodium-induced colitis by inhibiting ferroptosis. J. Dig. Dis..

[65] He H., Xu X., Yu Z., Xu F., Chen H. (2025). Regulation of Ferroptosis in Intestinal Epithelial Cells by Formononetin via the RXRA/PPARG Pathway. J. Interferon Cytokine Res..

[66] Niu R., Lan J., Liang D., Xiang L., Wu J., Zhang X., Li Z., Chen H., Geng L., Xu W. (2024). GZMA suppressed GPX4-mediated ferroptosis to improve intestinal mucosal barrier function in inflammatory bowel disease. Cell Commun. Signal..

[67] Huo C., Li G., Hu Y., Sun H. (2022). The Impacts of Iron Overload and Ferroptosis on Intestinal Mucosal Homeostasis and Inflammation. Int. J. Mol. Sci..

[68] Lin Y., Zhang Y., Wang X., Wang Y., Zhang A. (2026). YTHDF1 Drives Ferroptosis in Ulcerative Colitis via m^6^A-ACSL4 Stabilization. APMIS.

[69] Chen Y., Fan W., Lyu Y., Liao J., Zhou Y. (2025). METTL14 modulates the progression and ferroptosis of colitis by regulating the stability of m6A-modified GPX4. Eur. J. Med. Res..

[70] Liu C., Li J., Jin H., Zhao Q., Li F., Huang Z., Mei B., Gong W., Wang X., Han D. (2023). Colonic stem cell from severe ulcerative colitis maintains environment-independent immune activation by altering chromatin accessibility and global m^6^A loss. Life Med..

[71] Gu C., Wu J., Zhang W., Yao Y., Yan W., Yuan Y., Wang W., Shang A. (2022). Immune Infiltration of Ulcerative Colitis and Detection of the m6A Subtype. J. Immunol. Res..

[72] Motawi T.K., Shaker O.G., Amr G., Senousy M.A. (2024). RNA methylation machinery and m^6^A target genes as circulating biomarkers of ulcerative colitis and Crohn’s disease: Correlation with disease activity, location, and inflammatory cytokines. Clin. Chim. Acta.

[73] Lai Y., Liu J., Hu X., Zeng X., Gao P. (2025). N6-methyladenosine (m6A)-forming enzyme METTL3 controls UAF1 stability to promote inflammation in a model of colitis by stimulating NLRP3. Sci. Rep..

[74] Zhang H., Xu M. (2025). YTHDF1 activates FBW7 transcription by regulating m^6^A-dependent FOXO1 to facilitate inflammatory response in ulcerative colitis-like model. Autoimmunity.

[75] Ge X., Xue G., Ding Y., Li R., Hu K., Xu T., Sun M., Liao W., Zhao B., Wen C. (2023). The Loss of YTHDC1 in Gut Macrophages Exacerbates Inflammatory Bowel Disease. Adv. Sci..

[76] Xu X., Peng J., Wang N., Ocansey D.K.W., Zhang X., Mao F. (2024). hucMSC-Ex alleviates inflammatory bowel disease in mice by enhancing M2-type macrophage polarization via the METTL3-Slc37a2-YTHDF1 axis. Cell Biol. Toxicol..

[77] Leng X., Wang A., Wu Y., Wang H., Chen B., Long G., Yu P. (2026). RBM3-mediated m6A modification of KLF6 promotes ACSL4-driven ferroptosis in ulcerative colitis. Immunol. Cell Biol..

[78] Zheng Q., Qiu Y. (2025). Pannexin-1 Aggravates Inflammatory Bowel Disease via Unbalancing Macrophage Polarisation and Triggering Ferroptosis in Mice. Immunology.

[79] Shi H., Wei J., He C. (2019). Where, When, and How: Context-Dependent Functions of RNA Methylation Writers, Readers, and Erasers. Mol. Cell.

[80] Zhang Y., Hamada M. (2021). Identification of m^6^A-Associated RNA Binding Proteins Using an Integrative Computational Framework. Front. Genet..

[81] Sun S., Shen J., Jiang J., Wang F., Min J. (2023). Targeting ferroptosis opens new avenues for the development of novel therapeutics. Signal Transduct. Target. Ther..

[82] Wu X., Zhao L., Yu Z., Zhang K. (2024). Buddlejasaponin IVb Alleviates DSS-Induced Ulcerative Colitis through the Nrf2/GPX4 Pathway and Gut Microbiota Modulation. J. Agric. Food Chem..

[83] Peng G., Wang S., Zhang H., Xie F., Jiao L., Yuan Y., Ma C., Wu H., Meng Z. (2024). Tremella aurantialba polysaccharides alleviate ulcerative colitis in mice by improving intestinal barrier via modulating gut microbiota and inhibiting ferroptosis. Int. J. Biol. Macromol..

[84] Mayr L., Grabherr F., Schwarzler J., Reitmeier I., Sommer F., Gehmacher T., Niederreiter L., He G.W., Ruder B., Kunz K.T.R. (2020). Dietary lipids fuel GPX4-restricted enteritis resembling Crohn’s disease. Nat. Commun..

[85] Wen W., Xu Y., Qian W., Huang L., Gong J., Li Y., Zhu W., Guo Z. (2023). PUFAs add fuel to Crohn’s disease-associated AIEC-induced enteritis by exacerbating intestinal epithelial lipid peroxidation. Gut Microbes.

[86] Pan X., Zhu Q., Pan L.L., Sun J. (2022). Macrophage immunometabolism in inflammatory bowel diseases: From pathogenesis to therapy. Pharmacol. Ther..

[87] Ocansey D.K.W., Yuan J., Wei Z., Mao F., Zhang Z. (2023). Role of ferroptosis in the pathogenesis and as a therapeutic target of inflammatory bowel disease (Review). Int. J. Mol. Med..

[88] Xu S., He Y., Lin L., Chen P., Chen M., Zhang S. (2021). The emerging role of ferroptosis in intestinal disease. Cell Death Dis..

[89] Huang J., Zhang J., Ma J., Ma J., Liu J., Wang F., Tang X. (2022). Inhibiting Ferroptosis: A Novel Approach for Ulcerative Colitis Therapeutics. Oxidative Med. Cell. Longev..

[90] Shi J., Ji S., Xu M., Wang Y., Shi H. (2024). Selenium inhibits ferroptosis in ulcerative colitis through the induction of Nrf2/Gpx4. Clin. Res. Hepatol. Gastroenterol..

[91] Li J., Tian X., Liu J., Mo Y., Guo X., Qiu Y., Liu Y., Ma X., Wang Y., Xiong Y. (2022). Therapeutic material basis and underling mechanisms of Shaoyao Decoction-exerted alleviation effects of colitis based on GPX4-regulated ferroptosis in epithelial cells. Chin. Med..

[92] Hu S., Luo Y., Yang X., Zhou Z., Zhou F., Zhong L., Tan Y., Pei G., Tan Y. (2023). Shaoyao Gancao Decoction protects against dextran sulfate sodium-induced ulcerative colitis by down-regulating ferroptosis. J. Pharm. Pharmacol..

[93] Ji W., Zhang Y., Qian X., Hu C., Huo Y. (2024). Palmatine alleviates inflammation and modulates ferroptosis against dextran sulfate sodium (DSS)-induced ulcerative colitis. Int. Immunopharmacol..

[94] Li Y., Ma M., Wang X., Li J., Fang Z., Li J., Yang B., Lu Y., Xu X., Li Y. (2024). Celecoxib alleviates the DSS-induced ulcerative colitis in mice by enhancing intestinal barrier function, inhibiting ferroptosis and suppressing apoptosis. Immunopharmacol. Immunotoxicol..

[95] Jiang P., Zhai Z., Zhao L., Zhang K., Duan L. (2024). α-Lipoic acid alleviates dextran sulfate sodium salt-induced ulcerative colitis via modulating the Keap1-Nrf2 signaling pathway and inhibiting ferroptosis. J. Sci. Food Agric..

[96] Ru Y., Luo Y., Liu D., Huang Q., Zhou X., Linghu M., Luo X., Lv Z., Wu Y., Zhang H. (2024). Isorhamnetin alleviates ferroptosis-mediated colitis by activating the NRF2/HO-1 pathway and chelating iron. Int. Immunopharmacol..

[97] Deng B., Wang K., He H., Xu M., Li J., He P., Liu Y., Ma J., Zhang J., Dong W. (2025). Biochanin A mitigates colitis by inhibiting ferroptosis-mediated intestinal barrier dysfunction, oxidative stress, and inflammation via the JAK2/STAT3 signaling pathway. Phytomedicine.

[98] Li D., Xu F., Wang W., Jiang C., Cui W., Guan Z.A., Gu C. (2025). Nrf2/HO-1-dependent inhibition of ferroptosis underlies the antioxidant effects of 5-O-methylvisammioside in colitis. Front. Immunol..

[99] Wei Z., Hang S., Wiredu Ocansey D.K., Zhang Z., Wang B., Zhang X., Mao F. (2023). Human umbilical cord mesenchymal stem cells derived exosome shuttling mir-129-5p attenuates inflammatory bowel disease by inhibiting ferroptosis. J. Nanobiotechnol..

[100] Zhu Y., Qin H., Sun C., Shao B., Li G., Qin Y., Kong D., Ren S., Wang H., Wang Z. (2022). Endometrial Regenerative Cell-Derived Exosomes Attenuate Experimental Colitis through Downregulation of Intestine Ferroptosis. Stem Cells Int..

[101] Zhao Y., Yin W., Yang Z., Sun J., Chang J., Huang L., Xue L., Zhang X., Zhi H., Chen S. (2024). Nanotechnology-enabled M2 macrophage polarization and ferroptosis inhibition for targeted inflammatory bowel disease treatment. J. Control Release.

[102] Liu Y., Zhang J.T., Sun M., Song J., Sun H.M., Wang M.Y., Wang C.M., Liu W. (2025). Targeting ferroptosis in the treatment of ulcerative colitis by traditional Chinese medicine: A novel therapeutic strategies. Phytomedicine.

[103] Guo M., Du X., Wang X. (2024). Inhibition of ferroptosis: A new direction in the treatment of ulcerative colitis by traditional Chinese medicine. J. Ethnopharmacol..

[104] Wang Y., Hao Y., Yuan L., Tian H., Sun X., Zhang Y. (2024). Ferroptosis: A new mechanism of traditional Chinese medicine for treating ulcerative colitis. Front. Pharmacol..

[105] Yang Y., Hua Y., Zheng H., Jia R., Ye Z., Su G., Gu Y., Zhan K., Tang K., Qi S. (2024). Biomarkers prediction and immune landscape in ulcerative colitis: Findings based on bioinformatics and machine learning. Comput. Biol. Med..

